# Supplementary figures and images for: Pneumatic equiaxial compression device for mechanical manipulation of epithelial cell packing and physiology
Source: PLoS One. 2022 Jun 3;17(6):e0268570. doi: 10.1371/journal.pone.0268570 (PMC9165817; doi:10.1371/journal.pone.0268570)

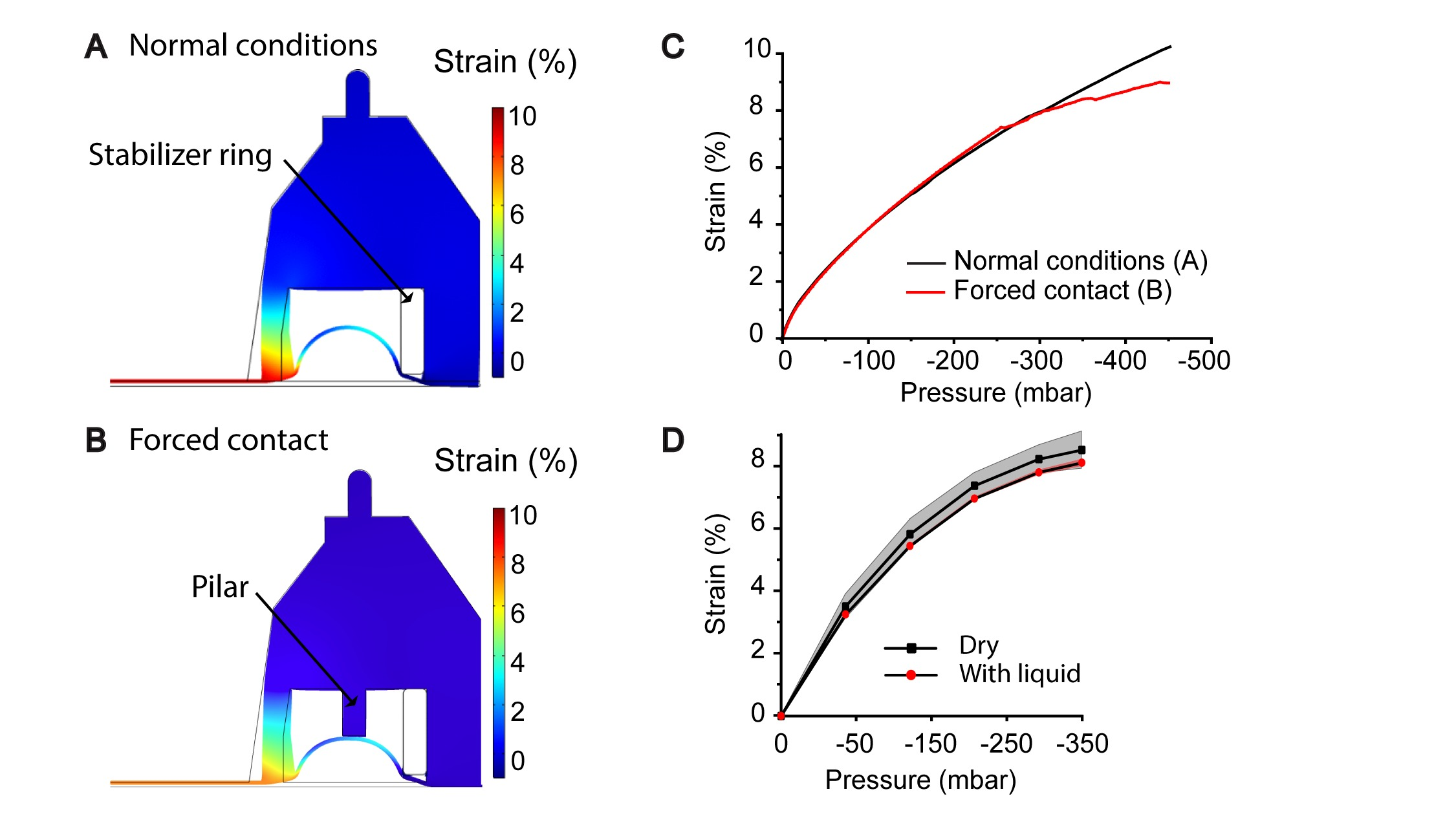

Supplement: S1 Fig — (A) Normal geometry used for simulations and a heat map of strain at -350 mbar. The stabilizer ring is marked with an arrow. (B) Artificial geometry where a pilar (marked with an arrow) is inserted below the vacuum chamber ceiling to force contact formation between the silicone membrane and the inner ceiling in the vacuum chamber. Heat map of strain at -350 mbar. (C) Simulated stress-strain data with the geometries presented in A and B. Forced contact causes strain to begin to saturate at -300 mbar. D) Comparison of strain measurements performed on dry devices (data from Fig 3D) and devices containing liquid. Strain data from liquid filled devices falls under the standard deviation region of devices measured dry. (TIF) [file pone.0268570.s001.tif]

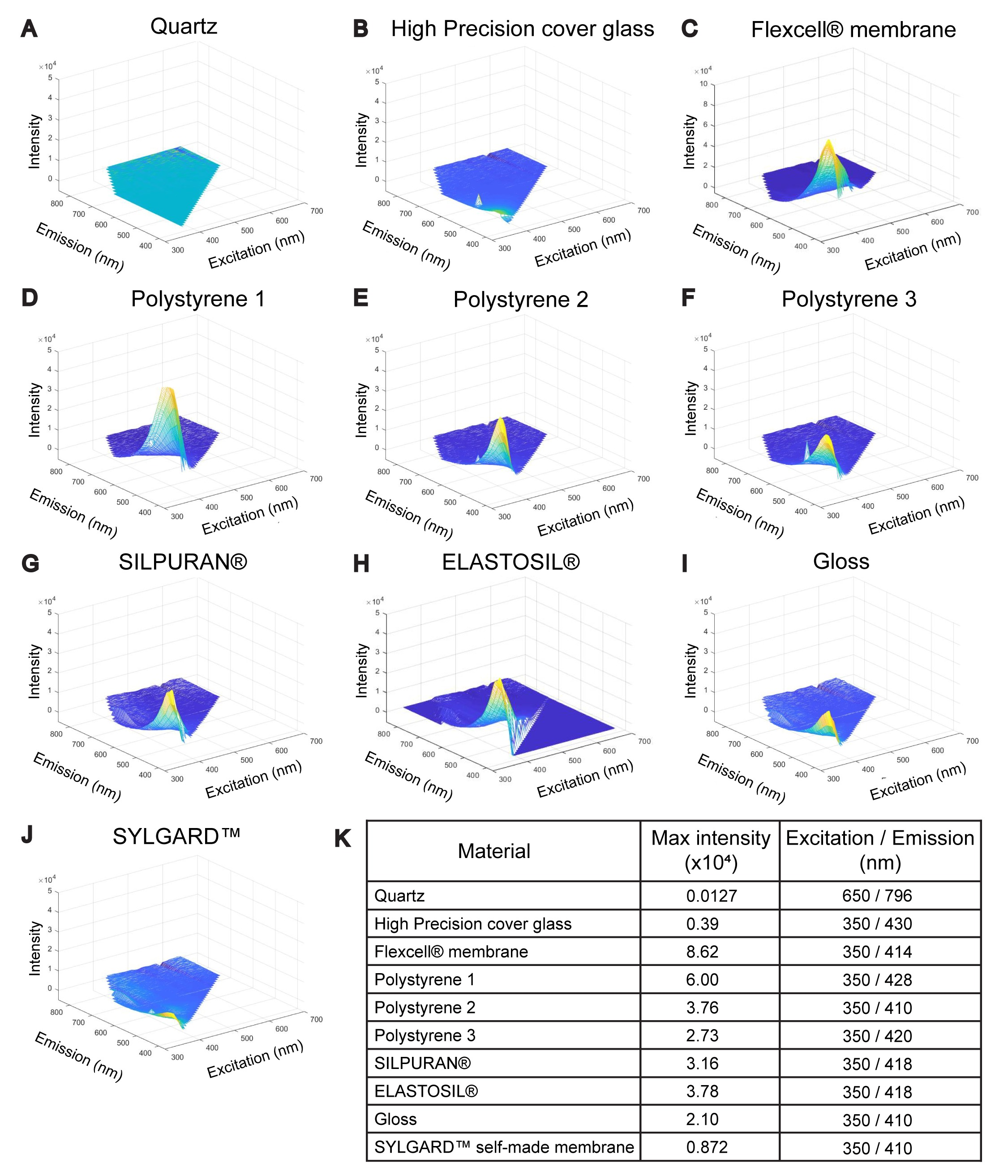

Supplement: S2 Fig — The excitation-emission-intensity plots for (A) Quartz, (B) High Precision cover glass, (C) Flexcell® membrane, (D-F) Polystyrene cell culture plastic, (G) SILPURAN®, (H) ELASTOSIL®, (I) Gloss, and (J) self-made SYLGARD™ membrane. (K) Table with maximum intensity values for each sample and the corresponding excitation and emission wavelengths. Note that Flexcell® membrane has a different intensity axis. (TIF) [file pone.0268570.s002.tif]
