# Supplementary material for: Pneumatic equiaxial compression device for mechanical manipulation of epithelial cell packing and physiology
Source: PLoS One. 2022 Jun 3;17(6):e0268570. doi: 10.1371/journal.pone.0268570 (PMC9165817; doi:10.1371/journal.pone.0268570)
Supplement: S1 Appendix — (DOCX) [file pone.0268570.s003.docx]

## S1 Appendix

## Manufacture of PDMS membrane

To make SYLGARD™ 184 membrane for the Full Width at Half Maximum (FWHM) measurements, PDMS (SYLGARD™ 184 Elastomer Kit, Dow, Midland, MI, USA) was mixed and degassed as described in materials and methods. The ~120 µm thick membrane was produced by spin coating (700 rpm, 30 sec) PDMS on a glass plate. The membrane was cured in 60°C for 10 h.

## Vacuum battery characterization

A pressure meter was attached between the device and vacuum battery. A pre-strain was applied (-800 mbar) and the vacuum battery was allowed to stabilize. The valve between the pressure battery and vacuum pressure controller was closed and the vacuum pressure controller removed so that the pressure in the device was maintained solely by the vacuum battery. Readings were taken from the pressure meter for 72 h to verify that the pressure was held. The mean leak rate was determined as 6.1 mbar/h. With this rate the vacuum battery maintains 99% of the pressure during the 30 min it took to transfer the setup to the microscope.

## Mini-incubator characterization

A device was filled with media, topped with a glass lid, inserted into the mini-incubator and maximum pre-strain was applied. The mini-incubator was placed on a heat plate set to 37° and 7 ml/min flow of 5% CO_2_, 19% O_2_, and 76% N_2_ gas was initiated. For control, the same media was pipetted on 2 wells of a 6-well plate and placed in a standard incubator. The samples were incubated O/N after which the pH of the media from the two conditions were measured. In control wells the pH was 7.270 and in the device 7.275. The 7 ml/min gas flow is therefore sufficient to maintain the pH of the cells cultured in a stretching device inside the mini-incubator.

## Autofluorescence measurements

Autofluorescence measurements were performed with FLS-1000 (Edinburgh Instruments, UK) spectrofluorometer, where an excitation-emission scan could be programmed. The excitation wavelengths were scanned in 10 nm steps from 350 nm to 650 nm, i.e., the spectrum usually used in fluorescent dyes. For each excitation wavelength, the emission was scanned in 2 nm steps with a 20 nm offset from the excitation wavelength until 850 nm. Commercial ELASTOSIL® (ELASTOSIL® FILM 2030, Wacker Chemie AG, Munich, Germany), SILPURAN® (SILPURAN® FILM 2030, Wacker Chemie AG, Munich, Germany) and Gloss (Gloss Sheeting, Specialty Manufacturing Inc., Saginaw, MI, USA) silicone membranes as well as self-made SYLGARD™ 184 (Dow) membrane were analysed. High Precision (Paul Marienfeld GmbH & Co. KG, Lauda-Königshofen, Germany) cover glass and quartz were used as negative controls, and three polystyrene petri dish plastics as positive controls.

As seen in S2 Fig, all samples except for quartz showed some emission at low excitation wavelengths. The cover glass had a small peak with 3.9 2x 10^3^ intensity. The three commercial silicone membranes including SILPURAN® had small peaks in x 10^4^ range whereas the self-made SYLGARD™ 184 membrane had slightly lower maximum intensity (9.72 2x 10^3^). All polystyrene plastics were also in the x 10^4^ range. These polystyrene culture dishes and especially the cover glass are commonly used in fluorescence imaging. As the maximum autofluorescence emission of the tested silicone membranes was in the same range as polystyrene plastic and cover glass, it can be assumed that the autofluorescence from the tested silicone membranes is insignificant.
